# FRESH AIR: an implementation research project funded through Horizon 2020 exploring the prevention, diagnosis and treatment of chronic respiratory diseases in low-resource settings

**DOI:** 10.1038/npjpcrm.2016.35

**Published:** 2016-06-30

**Authors:** Liza Cragg, Siân Williams, Niels H Chavannes

**Affiliations:** 1 International Primary Care Respiratory Group, Edinburgh, UK; 2 Department of Public Health and Primary Care, Leiden University Medical Centre, Leiden, The Netherlands

## Abstract

This protocol describes FRESH AIR, an implementation science project exploring how to improve the prevention, diagnosis and treatment of chronic lung diseases in contexts with limited healthcare resources. It consists of inter-related studies that take place in four countries that are part of the International Primary Care Respiratory Group’s (IPCRG) global network: Uganda, the Kyrgyz Republic, Vietnam and Greece. The project has been funded by the European Commission Horizon 2020 research programme and runs from October 2015 until September 2018.

## Background

Chronic respiratory diseases present a growing global burden of disease. Worldwide, about 210 million people have COPD, and asthma affects an estimated 300 million individuals.^1,2^ COPD is now the third leading cause of death worldwide.^3^ COPD and asthma are also major causes of morbidity due to persistent symptoms, reduced lung function and intermittent exacerbations that adversely affect functional status and quality of life. According to the World Health Organization (WHO), over 90% of COPD deaths and over 80% of asthma deaths occur in low- and middle-income countries (LMICs).^4^ Although prevalence data in LMICs are often inadequate, individual studies have revealed rates higher than in high-income countries. For example, a recent prospective cross-sectional observational study in rural Masindi, Uganda, found that the prevalence of spirometry-defined COPD in people older than 30 years was 16.2%. The prevalence was especially high (39%) in people aged 30–39 years.^5^

The link between exposure to smoke and lung diseases is well established. The Global Burden of Disease study 2013 identified air pollution (indoor and outdoor) as responsible for 10.1% of deaths worldwide in 2013, approaching the same impact as tobacco smoke, responsible for 11.2%.^6^ In 2014, the Lancet Respiratory Medicine Commission published a substantial review of the risks of lung disease from household air pollution (HAP) in low- and middle-income countries.^7^ It reported that a third of the world’s population used solid fuel derived from plant material (biomass) or coal for cooking, heating or lighting. These fuels are smoky, often used in an open fire or simple stove with incomplete combustion and poor ventilation, causing substantial HAP.

Tobacco kills around 6 million people worldwide every year. Although tobacco use is decreasing in many high-income countries, it is increasing in many LMICs. By the year 2030, 80% of deaths caused by tobacco use are expected to occur in LMICs.^8^ Although tobacco use continues to be the leading global cause of preventable death, there are proven, cost-effective means to combat this deadly epidemic.^9^

Acute respiratory infections are the leading cause of mortality in those aged under 5 years, with most deaths occurring in LMICs.^10^ According to recent findings, a significant proportion of children presenting with cough and/or difficult breathing in association with fast breathing have asthma syndrome rather than pneumonia.^11^ The failure to institute appropriate care for acute asthma may contribute to treatment failure, prolonged illness and mortality.^12^ Exposure to biomass smoke is associated with increased acute respiratory tract infections, pneumonia, asthma attacks and impaired lung function.^13,14^ Second-hand tobacco smoke is also associated with a wide range of poor maternal and child health outcomes including stillbirths, low-birth-weight babies and asthma attacks.^15^ Pregnancy outcomes such as low birth weight, neonatal death, premature labour and pre-eclampsia have been linked to HAP in epidemiological studies.^16^

Although the greatest burden of disease is experienced in LMICs, these countries are low-resource settings for healthcare that also experience significant challenges in implementing clinically effective and cost-effective interventions.^17^ There is growing recognition of the need to improve the translation of evidence into practice in low-resource settings.^18^ However, this requires understanding the implementation challenges experienced by healthcare systems in low-resource settings in applying the evidence for effective interventions for the prevention, diagnosis and treatment of chronic lung diseases, including a lack of awareness amongst policymakers, clinicians or the public about the health risks of tobacco and HAP.

This protocol presents the aim, concepts and methods of an innovative research project, FRESH AIR (Free Respiratory Evaluation and Smoke-exposure reduction by primary Health cAre Integrated gRoups), which addresses the need to prevent, diagnose and treat chronic lung diseases in low-resource settings. FRESH AIR, a 3-year project that began in October 2015, is funded through Horizon 2020. It builds on data, knowledge and experience gained by the International Primary Care Respiratory Group (IPCRG) from earlier FRESH AIR initiatives in low-resource settings.^5,19^

The FRESH AIR project is underpinned by four principles:

- Action to improve lung health needs to take place along a continuum encompassing awareness raising, prevention, diagnosis, treatment and support for patients and their families. In real-life practice, there are complex interactions between these that can result in barriers and facilitators of successful implementation.
- Promoting and protecting children’s lung health to prevent early mortality and to ensure that they can develop healthy lungs is essential at every stage of this continuum.
- For healthcare workers to implement interventions, they must see them as clinically important and have confidence in using them.^20^ Therefore, it is essential that practising clinicians have a leading role in designing, teaching and supporting the interventions.
- Primary care engagement is essential in tackling the current and future burden of non-communicable diseases, including chronic respiratory diseases.^21^ Primary care, no matter how it is set up, takes a holistic approach and applies generalist expertise to diagnose and manage people who may have multi-morbidity as opposed to treating single diseases. It also sees patients in the context of their families, homes and communities.

## Aims

The overall aim of the FRESH AIR project is to improve health outcomes for people at risk of, or suffering from, chronic lung diseases in low-resource settings by developing capacity for implementation of evidence-based interventions for prevention, diagnosis and treatment in these contexts. The project has seven specific objectives:

1. To identify the specific factors that influence the implementation of evidenced-based interventions in the prevention and treatment of non-communicable lung diseases in community settings.
2. To explore which awareness-raising approaches are most effective in motivating behaviour change in tobacco consumption and HAP exposure and to evaluate the feasibility, acceptability and effectiveness of HAP reduction interventions in selected communities.
3. To provide access to smoking cessation support by adapting successful evidence-based very brief advice (VBA) interventions.
4. To test the feasibility and acceptability of methods for diagnosing COPD using innovative spirometry in these four countries.
5. To test the feasibility and acceptability of pulmonary rehabilitation (PR) as a low-cost treatment for obstructive lung disease.
6. To test how to best reduce children’s respiratory symptoms and the risk of lung damage by exploring the feasibility, acceptability and optimal organisation of interventions designed to raise awareness of the damaging effects of exposure to tobacco smoke and HAP during pregnancy and infancy, and to improve diagnosis and treatment of children aged under 5 years presenting to primary care with respiratory symptoms.
7. To generate new knowledge, innovation and scalable models that ensure equitable access and to support their implementation through proactive dissemination.

## Methods

The project consists of inter-related studies that are designed to achieve these objectives. The studies will be carried out in four countries that are part of the International Primary Care Respiratory Group’s global network: Uganda, the Kyrgyz Republic, Vietnam and Greece. Each of these is a low-resource setting with high levels of tobacco consumption and population groups exposed to HAP. These countries also present a range of different implementation challenges because they are countries with diverse demographic, geographic, economic, health system and cultural characteristics.

The studies will use a range of implementation science methodologies to adapt and test innovative ways to implement the evidence. They will explore implementation science research questions, including what works, for whom and under what contextual circumstances and how to ensure scalability of effective interventions in ways that are accessible and equitable in low-resource settings. Details of each study and its research questions and methods are given in Table 1.

Patients, community groups, healthcare workers, policymakers and other stakeholders will be involved through Stakeholder Engagement Groups in each of the four countries in which FRESH AIR project activities take place. They provide input on local priorities and other contextual factors that are used in the detailed design of interventions.

The research included in the FRESH AIR project will be reported using the Standards for reporting implementation studies of complex interventions (StaRI).^22^ This includes a checklist of items to be included in reporting implementation studies and fits within the suite of EQUATOR reporting guidelines.

For management and implementation purposes, the studies are arranged in five work packages, with two supporting work packages. Each work package has defined objectives, tasks and deliverables, which are described in Supplementary Appendix 3.

## Discussion

The evidence base for clinically and cost-effective prevention, diagnosis and treatment of chronic respiratory disease, including for smoking cessation, is well developed for populations in high-income countries. However, there is insufficient evidence about the specific risk factors experienced by communities in LMICs and low-resource settings and the role of age and gender in risk exposure. Extrapolations from high-income countries to LMICs are particularly prone to errors about the balance of risk caused by indoor and outdoor air pollution.^23^ Furthermore, exposure to risk factors is influenced by local economic, social, cultural and other contextual factors that need to be understood in order to develop appropriate prevention and management strategies. In addition, diagnosis and treatment of lung disease in low-resource settings is hampered by barriers including poor public awareness of lung disease and its risk factors, lack of knowledge and engagement of policymakers, limited access to trained healthcare professionals, diagnostic facilities and treatment options. Finally, low-resource settings are seriously under-represented in current research into lung diseases. For example, a recent study on tobacco use found that only 4% of randomised controlled trials included in systematic reviews and 2% of on-going trials were performed in LMICs, even though these countries represented 70% of the mortality related to tobacco use.^24^

The FRESH AIR project will address these points through implementation science studies conducted in four countries with diverse demographic, geographic, economic and cultural characteristics: Uganda, the Kyrgyz Republic, Vietnam and Greece. Because of their different characteristics, these countries present a range of different implementation challenges that will enable the studies to generate generalisable conclusions of relevance to many more countries globally. This new knowledge will be widely disseminated nationally, regionally and internationally, ensuring the scale-up of interventions demonstrated as successful by the project and global impact of research findings. In doing so, the FRESH AIR project will have impacts at four levels:

1. Public health policy: by providing evidence, information and support for decision-making and improving understanding and knowledge of the links between risk factors, interventions and health outcomes.
2. Healthcare provision for individuals and populations: by developing and adapting evidence-based prevention, diagnostic and treatment models and generating new knowledge on implementation.
3. Professional awareness and skills: by teaching healthcare workers and developing new feasible and scalable teaching models.
4. Public perceptions and opinions: by developing and testing models that increase awareness and motivation for behaviour change and generating new knowledge on these.

**Figure 1:**
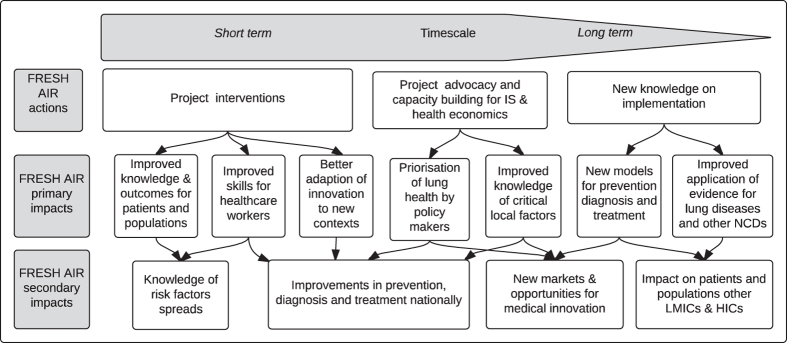
FRESH AIR impact map.

These can be divided into primary impacts, which happen as a direct result of FRESH AIR activities, and secondary impacts, which happen as a result of the increased knowledge and capacities the FRESH AIR project generates. The impacts will be achieved over different time scales. They are mapped in Figure 1.

The FRESH AIR project is delivered by a consortium of 14 organisations from 9 countries made up of some of the leading university hospitals in EU member states and the United States and policy experts and international healthcare societies that specialise in lung disease and smoking cessation. The consortium also includes healthcare providers, policymakers and implementers from the four countries in which project activities take place (see Supplementary Appendix 1 for members). The Consortium is supported by a Scientific Advisory Committee made up of leading clinicians, scientists and researchers with experience in implementation science, lung disease, tobacco dependence, clinical care and service delivery in low-resource settings (see Supplementary Appendix 2 for members). The International Primary Care Respiratory Group (IPCRG) maintains a public website (http://www.theipcrg.org/freshair) to enable emerging findings to be shared widely and freely, and private pages for the logging of contextual data, as recommended in the latest guidance on implementation science reporting.^24^

## The FRESH AIR Group

Meerim Akmatalieva, The Kyrgyz Republic; Emilov Berik, The Kyrgyz Republic; Antonios Bertsias, Greece; Evelyn Brakema, The Netherlands; Dennis Burges, USA; Vasiliki-Eirini Chatzea, Greece; Niels Chavannes, The Netherlands; Jaime Correia de Sousa, Portugal; Liza Cragg, France; Matty Crone, The Netherlands; Irene Ferrario, UK; Liz Grant, UK; Christina Gratziou, Greece; Catherine Hartmann, Belgium; Nicholas S. Hopkinson, UK; Rupert Jones, UK; Ben Hedrick, USA; Sharon Winnie Kiche, USA; Bruce Kirenga, Uganda; Jesper Kjærgaard, Denmark; Christos Lionis, Greece; Maamed Mademilov, The Kyrgyz Republic; David Martenson, USA; Andy McEwen, UK; Rebecca Nantada, Uganda; Grace Ndeezi, Uganda; Vinh Nhu Nguyen, Vietnam; Mattijs Numans, The Netherlands; Sophia Papadakis, Greece; Hilary Pinnock, UK; Maarten J Postma, The Netherlands; Anja Poulsen, Denmark; Pippa Powell, UK; Ria Reis, The Netherlands; Sundeep Salvi, India; Saltanat Shabykeeva, The Kyrgyz Republic; Dimitra Sifaki, Greece; Sally Singh, UK; Talant Sooronabaev, The Kyrgyz Republic; Jacob Sont, The Netherlands; Jim Stout, USA; Marianne Stubbe Østergaard, Denmark; Aizhamal Tabyshova, The Kyrgyz Republic; Le Thi Tuyet Lan, Vietnam; Tuan Tran Diep, Vietnam; Ioanna Tsiligianni, Greece; James Tumwine, Uganda; Job FM van Boven, The Netherlands; Rianne van der Kleij, The Netherlands; Thys van der Molen, The Netherlands; Frederik van Gemert, The Netherlands; Louise Warren, USA; Siân Williams, UK; Savithri W Wimalasekera; Sri Lanka; Arzu Yorgancıoğlu, Turkey; Karen Zeribi, USA.

## Figures and Tables

**Table 1:** FRESH AIR studies

*Study area of each objective*	*Research questions*	*Methods*
Objective 1: prevalence, exposure and burden	• What is the expected and observed burden of chronic respiratory diseases and exposure to risk factors, including HAP and tobacco consumption in each of the four countries?	• Quantitative analysis of existing data
		
Objective 1: beliefs and perceptions of respiratory symptoms and their causes	• What beliefs, perceptions and behaviours are observed about respiratory symptoms and their causes?	• Participative workshops • Interviews
		
Objective 1: critical factors for implementation	• What are the critical factors for the successful implementation of evidence-based interventions to reduce HAP and tobacco smoke exposure?	• Systematic review of literature
		
Objective 2: action research on awareness raising	• What are the factors that influence awareness of, and attitude to, risks of HAP and tobacco among healthcare workers and the public? • How can communities be motivated to change their behaviour to reduce their exposure to smoke from HAP and tobacco?	• Development and testing of resources using Plan Do Study Act (PDSA) cycles^25^ • Training for healthcare workers • Household questionnaires • Patient questionnaires
		
Objective 2: reducing exposure to household air pollution	• What are the local barriers to accessing clean fuel? • What opportunities exist to improve access to clean fuels and how can these be maximised?	• Training for healthcare workers • Before and after awareness questionnaires • Monitoring 30 households on indoor pollution before and after intervention
		
Objective 3: very brief advice training for healthcare workers	• Who are the best placed healthcare workers to provide very brief advice in contexts where access to health care is limited? • What are the obstacles and facilitators for these healthcare workers in these contexts to provide very brief advice?	• Data collection through questionnaires • Mapping of service provision • Interviews with healthcare professionals • Interviews with patients
		
Objective 4: improving diagnostics for COPD	• What is the acceptability and feasibility of using the SpiroSmart smart phone spirometer in low-resource settings? • How can healthcare workers be supported to administer and interpret spirometry for improved diagnosis?	• Feasibility study • Data collection through questionnaires and/or focus groups about user experience • Pilot training with follow-up • Interviews with healthcare professionals
		
Objective 5: pulmonary rehabilitation feasibility study	• How can pulmonary rehabilitation (PR) programmes be set up in low-resource settings? • What are the community’s attitudes to exercise programmes and what socially acceptable and equitable opportunities are there to increase the exercise capacity of people at risk of chronic lung disease? • Are IT-based home PR methods applicable in remote settings?	• Feasibility study • Semi-structured interviews • Focus groups with practitioners, patients and other key stakeholders
		
Objective 6: midwife-led smoke reduction study	• Is a HAP and tobacco smoke reduction education programme delivered by midwives and village HC teams feasible and acceptable? • Does it reduce exposure to particulate matter and carbon monoxide? • Does it improve a range of health outcomes in pregnancy and infancy including respiratory outcomes at the age of 6 months?	• Pilot cluster randomised controlled trial
		
Objective 6: research on terms, concepts and treatment practices for childhood asthma	• What are the concepts and terms used by carers, healthcare providers and local experts (e.g., traditional healers) for long-term coughing, asthma/wheeze and ARI? • What are the treatment practices for management of long-term cough and ARI in children aged under 5 years in health centres and by local experts?	• Qualitative research, including interviews with parents, healthcare professionals and traditional healers
		
Objective 6: asthma and acute respiratory infection study	• What is the feasibility, acceptability and optimal organisation for the roll-out of the findings of a hospital-based intervention using asthma treatment for children presenting with ARI in primary care in rural settings to reduce infant mortality?	• PDSA cycles • Observation • Cost and logistics analysis of supply of medicines • Qualitative research with healthcare workers and carers
		
Objective 7: cost-effectiveness reviews	• Which interventions are most cost-effective in low-resource settings? • How many people in the population to be studied have COPD/asthma/the relevant condition? • How many people could benefit from the intervention? • What is the cost of providing this intervention for these people?	• Data analysis using STAR (socio-technical allocation of resources) approach^26^ • Facilitated workshops for stakeholders using STAR approach
		
Objective 7: capacity building in implementation science to key stakeholders	• What are the local obstacles and facilitators to translating evidence into practice including context, organisation, professional issues and availability of interventions?	• Participative workshops • Interviews with stakeholders

## Notes

NHC is an Associate Editor of *npj Primary Care Respiratory Medicine*, but was not involved in the editorial review of, nor the decision to publish this article.
