# Supplementary material for: FRESH AIR: an implementation research project funded through Horizon 2020 exploring the prevention, diagnosis and treatment of chronic respiratory diseases in low-resource settings
Source: NPJ Prim Care Respir Med. 2016 Jun 30;26:16035–. doi: 10.1038/npjpcrm.2016.35 (PMC4928382; doi:10.1038/npjpcrm.2016.35)
Supplement: Supplementary Appendix 1 [file npjpcrm201635-s1.doc]

***Appendix 1: FRESH AIR Consortium members***

| ***Institution*** | ***Lead*** | ***Country*** | ***Website link*** |
| --- | --- | --- | --- |
| Leiden University Medical Centre | Prof Niels Chavannes | Netherlands | www.lumc.nl |
| International Primary Care Respiratory Group | Ms Siân Williams | UK | www.theipcrg.org |
| Makerere University College of Health Sciences | Dr Bruce Kirenga | Uganda | http://www.mak.ac.ug |
| Ministry of Health Kyrgyz Republic | Prof Talant Sooronbaev | Kyrgyz Republic | www.med.kg |
| University of Medicine & Pharmacy, Ho Chi Minh City | Dr An Le Pham | Vietnam | http://bacsigiadinhvietnam.org |
| University of Crete | Prof Christos Lionis | Greece | www.fammed.uoc.gr |
| ARTEG | Prof Maarten Postma | Netherlands | http://www.rug.nl/staff/m.j.postma/ |
| European Lung Foundation | Ms Pippa Powell | UK | www.europeanlung.org |
| University of Washington | Prof James Stout | USA | http://washington.edu/medicine/pediatrics |
| National Centre for Smoking Cessation and Training | Dr Andy McEwen | UK | www.ncsct.co.uk |
| University of Groningen | Dr Frederik van Gemert | Netherlands | www.umcg.nl |
| University of Copenhagen | Dr Marianne Stubbe Ostergaard | Denmark | www.ifsv.ku.dk/afdelinger/almen_medicin/ |
| European COPD Coalition | Ms Catherine Hartmann | Belgium | www.copdcoalition.eu |
| Plymouth University | Dr Rupert Jones | UK | www.plymouth.ac.uk/ |
