# Supplementary material for: FRESH AIR: an implementation research project funded through Horizon 2020 exploring the prevention, diagnosis and treatment of chronic respiratory diseases in low-resource settings
Source: NPJ Prim Care Respir Med. 2016 Jun 30;26:16035–. doi: 10.1038/npjpcrm.2016.35 (PMC4928382; doi:10.1038/npjpcrm.2016.35)
Supplement: Supplementary Appendix 2 [file npjpcrm201635-s2.doc]

***Appendix 2: FRESH AIR Scientific Advisory Committee members***

- ***Dr Hilary Pinnock***, ***Co-Chairperson,*** Reader at the University of Edinburgh, Centre for Population Health Sciences
- ***Professor Thys Van Der Molen*** ***Co-Chairperson,*** Professor of Primary Care Respiratory Medicine, University of Groningen Medical Center.
- ***Professor Arzu Yorgancıoğlu***, head of the Department of Pulmonology at Celal Bayar University in Manisa, Turkey.
- ***Dr Nicholas S. Hopkinson***, Clinical Senior Lecturer, National Heart and Lung Institute, Imperial College, London; clinical lead for COPD, Royal Brompton Hospital, London.
- ***Professor Christina Gratziou***, Associate Professor for Pulmonary Medicine and Critical Care, Medical School of the National and Kapodistrian University, Athens; Head of the Research Center of Tobacco Control and Smoking Cessation Clinic, University Pulmonary Department of Athens.
- ***Dr Liz Grant***, Assistant Principal, Global Health; Director of the Global Health Academy, University of Edinburgh.
- ***Associate Professor Le Thi Tuyet Lan,*** Chairwoman of Ho Chi Minh City Respiratory Society, Head of Respiratory Care Center; Head of Functional Explorations Department, Hospital of Medical and Pharmacy University, Ho Chi Minh Medical and Pharmacy University
- ***Professor Sally Singh,*** Head of Pulmonary and Cardiac Rehabilitation, University Hospitals of Leicester NHS Trust
- ***Assoc Prof Jaime Correia de Sousa,*** Associate Professor, School of Health Sciences, University of Minho
- ***Dr Sundeep Salvi,*** Director of the Chest Research Foundation, Pune
- ***Associate Professor Savithri W Wimalasekera*** Department of Physiology, Faculty of Medical Sciences, University of Sri Jayewardenepura
