# Supplementary material for: FRESH AIR: an implementation research project funded through Horizon 2020 exploring the prevention, diagnosis and treatment of chronic respiratory diseases in low-resource settings
Source: NPJ Prim Care Respir Med. 2016 Jun 30;26:16035–. doi: 10.1038/npjpcrm.2016.35 (PMC4928382; doi:10.1038/npjpcrm.2016.35)
Supplement: Supplementary Appendix 3 [file npjpcrm201635-s3.doc]

Appendix 3: Overview of FRESH AIR work packages

| ***Work package*** | ***Objectives*** | ***Tasks*** | ***Deliverables (Month due)*** | ***Lead*** |
| --- | --- | --- | --- | --- |
| WP 1: Coordination | - To ensure efficient and effective operational project management, communication with the EC and cross-work package coordination, communication and learning. - To provide financial and legal control. | - Operational management and coordination; - Financial and legal control. | - Consortium Agreement (M1) | Prof Niels Chavannes (LUMC) |
| WP 2: Developing capacity for implementation science | - To provide training and education to policy makers, researchers and other stakeholders to increase capacity and demand for research using implementation science. - To provide methodological support for WPs leads, country leads and other project participants. - To undertake cost-effectiveness evaluation of interventions. | - Develop and deliver capacity building in implementation science to key stakeholders; - Provide methodological support for WPs; - Undertake cost-effectiveness reviews using the STAR approach. | - Cost-effectiveness analyses of WP 3, 4, 5, and 6. (M12) - Analysis of the health economic impact of lung disease for each country (M18) - Submission of a paper capturing the learning (M30) | Prof Jim Stout (UW) |
| WP3: Making the case for action | - To identify the expected and observed burden of lung diseases and exposure to risk factors, including HAP and tobacco consumption, in each of the four countries. - To explore beliefs and perceptions of respiratory symptoms and their causes and resultant clinical and patient behaviours in each of the four countries. - To identify critical factors hampering successful implementation of evidence-based interventions to prevent and treat lung diseases and engage the community to want to find solutions. - To use this research to raise awareness of the scale and importance of the problem of lung diseases and exposure to risk factors, including HAP and tobacco consumption, amongst policy makers and other key stakeholders and make the case for increasing capacity to address these. | - Quantitative analysis of the prevalence (expected and observed) of lung diseases and exposure to risk factors in the selected countries; - Qualitative research on beliefs and perceptions of respiratory symptoms and their causes and resultant clinical and patient behaviours in the selected countries; - Systematic review of literature on critical factors for the successful implementation of evidence-based interventions to prevent lung diseases in LMICs, supplemented by consensus building using the Delphi Technique - State-of-the-art knowledge base; - Advocacy. | - Paper on the quantitative research on the prevalence and risk factors (M18) - Paper on the qualitative research into beliefs and perceptions of respiratory symptoms (M20) - Full analysis and knowledge base (M24) - D3.5 Materials summarising key messages and promoting use of the knowledge base (M24) | Prof Niels Chavannes (LUMC) |
| WP4: Preventing lung disease by reducing exposure to HAP and tobacco | - To promote behaviour change by designing and testing interventions to raise community awareness of the damaging impacts of tobacco and HAP caused by burning biomass fuel in specific areas of the four countries. - To evaluate the feasibility, acceptability and effectiveness of proven household air pollution reduction interventions in selected communities in four countries. | - Action research on awareness raising in four countries; - Participative research on reducing exposure to HAP caused by biomass fuel use. | - Paper on awareness raising action research (M34) - Paper on participative research on reducing exposure to HAP (M34) | Dr Frederik van Gemert (UMCG) |
| WP5: Improving diagnosis and treatment | - To explore how health care workers can be supported to use spirometry for improved diagnosis of COPD. - To identify how Very Brief Advice on smoking cessation can be provided in LMICs. - To provide resources for training health care workers to provide very brief smoking cessation advice in four countries. - To identify how pulmonary rehabilitation programmes can be provided in LMICs. - To carry out pilot interventions training teams to provide pulmonary rehabilitation (PR). | - Improving diagnostics for COPD; - The adaptation of the Very Brief Advice smoking cessation module for healthcare workers; - Deliver training for healthcare workers in PR and the testing of the feasibility of low cost and low technology based alternative approaches to PR. | - Adapted VBA training modules (M24) - Delivery of spirometry training (M18) - Training for healthcare workers in PR delivered in three countries with a transferable training module and resources (M24) - Paper on adaption of VBA submitted to a scientific journal (M30) - Paper on PR (M30) - Follow-up analysis of spirometry training (M34) - Paper exploring the acceptability, feasibility, and end-user experience of SpiroSmart (M36) | Dr Rupert Jones (PU) |
| WP6: Protecting and improving lung health in infancy and childhood | - To explore the feasibility and acceptability of an education intervention aimed at reducing exposure to HAP in pregnancy. - To improve the diagnosis and treatment of childhood respiratory illnesses accurately including differentiation between infection and exacerbation of chronic obstructive disease. - To explore the feasibility, acceptability and optimal organisation for a low budget treatment model for ARI and asthma/wheeze treatment in primary care in rural settings. - To increase awareness of the damaging effects of exposure to biomass smoke during infancy and childhood. | - Midwife-led smoke reduction study; - Research on traditional concepts and treatments for childhood cough and wheeze; - Asthma and acute respiratory infection study; - Comparative analysis and policy review. | - Paper on research into existing perceptions of and practices around respiratory symptoms in children (M24) - Paper on asthma and acute respiratory infection study (M33) - Paper on midwife led smoke reduction study (M33) - Comparative country analysis (M34) | Dr Marianne Stubbe Østergaard (UCPH) |
| WP7: Maximising and spreading impact | - To ensure the active engagement of stakeholders in four countries (Vietnam, Uganda, Kyrgyz Republic, Greece) in the project, with a view to embedding the project interventions into policy. - To develop accessible communication materials. - To disseminate the knowledge, evidence and learning generated by the project to policy makers, healthcare providers, researchers and others in the EU and globally. - To ensure scientific excellence through a Scientific Advisory Committee. | - Stakeholder engagement; - Developing accessible communication material; - Proactive communication and dissemination; - Convening the Scientific Advisory Committee. | - Communication and Dissemination Strategy (M3) - User friendly information tools for health care workers (M6, 12, 18, 24, 30) | Siân Williams (IPCRG) |
